# Artifact-free holographic light shaping through moving acousto-optic holograms

**DOI:** 10.1038/s41598-021-00332-4

**Published:** 2021-10-28

**Authors:** Dorian Treptow, Raúl Bola, Estela Martín-Badosa, Mario Montes-Usategui

**Affiliations:** 1 Optical Trapping Lab – Grup de Biofotònica, Departament de Física Aplicada, Facultat de Física, Universitat de Barcelona, Martí i Franquès 1, 08028 Barcelona, Spain; 2 Institut de Nanociència i Nanotecnologia (IN2UB), 08028 Barcelona, Spain

**Keywords:** Optoelectronic devices and components, Displays, Photonic devices

## Abstract

Holographic light modulation is the most efficient method to shape laser light into well-defined patterns and is therefore the means of choice for many intensity demanding applications. During the last two decades, spatial light modulators based on liquid crystals prevailed among several technologies and became the standard tool to shape light holographically. But in the near future, this status might be challenged by acousto-optic deflectors. These devices are well known for their excelling modulation rates and high optical power resilience. But only few scattered precedents exist that demonstrate their holographic capabilities, despite the many interesting properties that they provide. We implemented a holographic acousto-optic light modulation (HALM) system, that is based on displaying holograms on acousto-optic deflectors. We found that this system can eliminate the ubiquitous coherent artifacts that arise in holography through the inherent motion of acousto-optic holograms. That distinguishes our approach from any other holographic modulation technique and allows to reconstruct intensity patterns of the highest fidelity. A mathematical description of this effect is presented and experimentally confirmed by reconstructing images holographically with unprecedented quality. Our results suggest that HALM promotes acousto-optic deflectors from highly specialized devices to full-fledged spatial light modulators, that can compete in a multitude of applications with LC-SLMs. Especially applications that require large optical output powers, high modulation speeds or accurate gray-scale intensity patterns will profit from this technology. We foresee that HALM may play a major role in future laser projectors and displays, structured illumination microscopy, laser material processing and optical trapping.

## Introduction

Programmable laser pattern generators based on spatial light modulators (SLMs) are key tools in a broad range of applications in optics and photonics. SLMs modulate the amplitude and phase of a wavefront to generate well defined two-dimensional patterns of laser light for the use in material processing, additive manufacturing, fluorescence microscopy, optical micro-manipulation and lithography, to name only a few^1–6^. Holographic light modulation with phase optimized holograms is the most efficient method to form a laser beam into a desired spatial intensity distribution, because the phase modulation redirects the light (instead of blocking it) to form areas of different light intensity. But unfortunately, the coherency of laser light naturally results in coherent artifacts akin to speckle, that degrade holographically reconstructed patterns severely. These coherent artifacts appear because the point spread functions of neighboring image points overlap and interfere constructively or destructively according to their optical phase difference^7^. Commonly, the optical phases are distributed randomly as a consequence of hologram optimization methods. This results in a pattern of randomly distributed high and low intensity areas that degrade the reconstructed images with a noise-like granular pattern, which is also referred to as holographic speckle.

Many different methods that reduce holographic speckle have been developed in response to this need^8^. However, the present solutions either have a limited level of effectiveness or they come with a drawback, which negatively affects crucial performance parameters of the system such as the light efficiency, the pattern fidelity, or the reconstruction speed. One category of speckle reduction techniques is based on constraining the phase of the reconstructed pattern to reduce the phase difference between neighboring points^9^. That reduces the speckle contrast, but at the same time, the diffraction efficiency reduces because the image phase cannot be freely optimized. An alternative solution is a pixel separation technique that decomposes an image into sparse arrays of spatially isolated points that are successively reconstructed to form the final image by integration over time^10^. On the one hand, the sparseness prevents the interference of simultaneously reconstructed image points and therefore avoids coherent artifacts. But on the other hand, the time required to reconstruct a pattern increases proportional to the number of sparse patterns that need to be integrated, and so the reconstruction speed reduces with this method. Light sources with low spatial or temporal coherence where also discussed as possible solutions to reduce holographic speckle^11,12^. That, however, impairs the resolution and contrast of a holographically reconstructed pattern, because phase holograms form images through interference and rely on a high degree of light coherence. Another class of methods is based on generating uncorrelated holographic speckle patterns successively over time, which average out when integrated by a detector. A variation of the speckle pattern is obtained by changing the optical phase distribution of the reconstructed pattern, what can be achieved e.g. by reconstructing the same target pattern with different holograms or by circular shifting the same hologram on the SLM^13,14^. The main disadvantage of these methods is that the speckle contrast *c* reduces only slowly with the number *N* of averaged uncorrelated patterns (i.e. *c* ∝ 1/√*N*), so that a trade-off between projection speed and speckle contrast is made.

**Figure 1:**
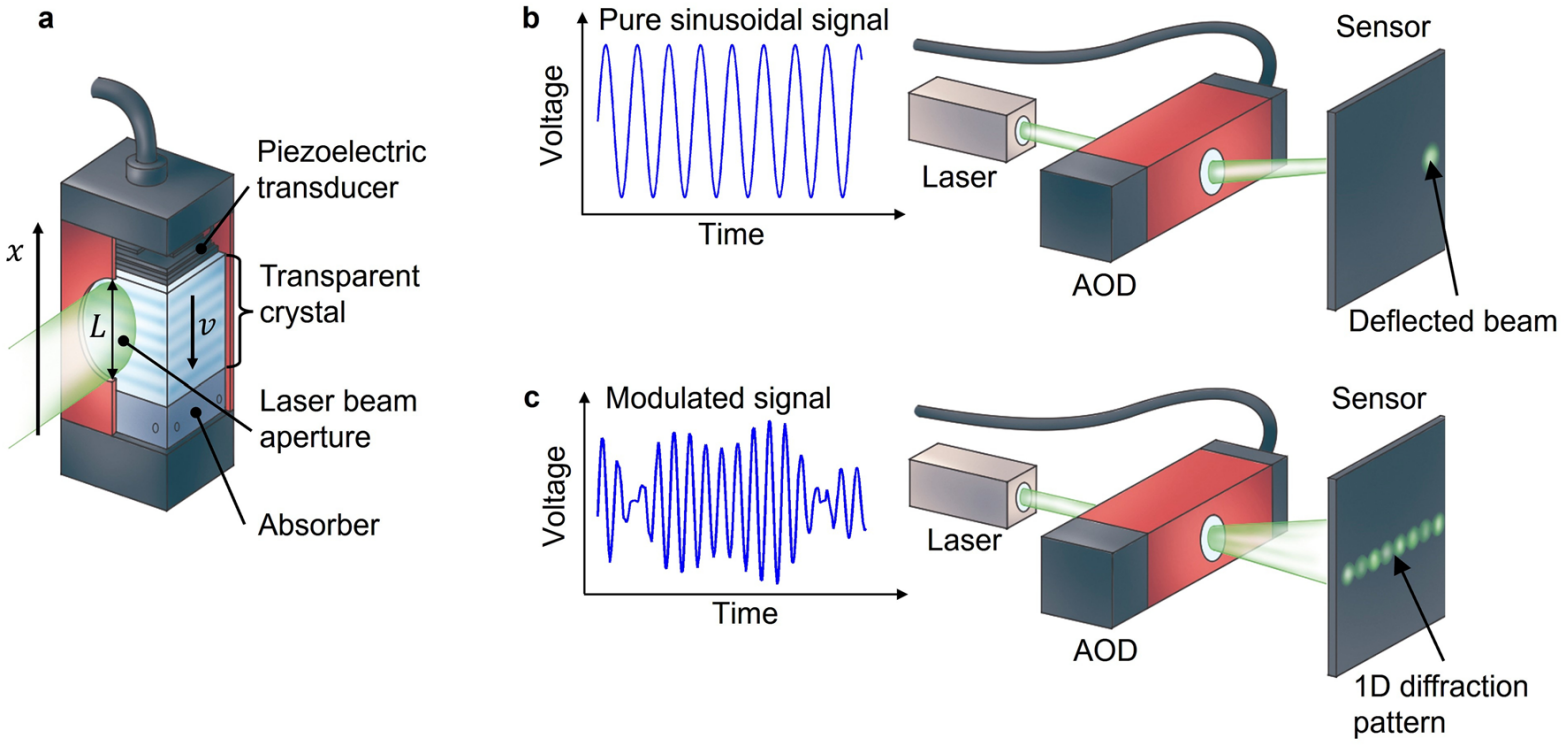
Acousto-optic light modulation. (**a**) Working principle of an acousto-optic deflector. An electrical driving signal acts on the piezo-electric transducer, which in response launches an acoustic wave in the attached transparent crystal. The resulting variation of the refractive index along the direction of acoustic propagation (*v*) leads to a spatial phase modulation of the transmitted laser beam. An absorber prevents reflections of the acoustic wave at the end of the crystal. (**b**) A sinusoidal driving signal deflects the transmitted laser beam. (**c**) An amplitude and phase modulated driving signal can generate a one-dimensional diffraction pattern.

Nevertheless, a light-modulating device exists that naturally introduces a time variant optical phase in reconstructed images and therefore holds the potential for an inherent speckle reduction mechanism, without compromising its modulation performance: the acousto-optic deflector (AOD). AODs are one-dimensional light modulating devices that shape light through a volatile diffraction grating that is aroused by an acousto-optic wave travelling in a transparent medium (see Fig. 1a). The acoustic wave is controlled via an electronic driving signal that acts on a piezo-electric transducer, which in turn is connected to this medium. In the vast majority of applications, the driving signals are sinusoids that deflect a laser beam at a certain angle according to the signal frequency (see Fig. 1b). That allows an extremely fast beam scanning at kilohertz rates, which found many applications in material processing, microscopy and optical micromanipulation^15–19^. But it has also been demonstrated in few precedents, which are scattered over decades and various fields of optics and photonics, that complexly modulated signals allow AODs to change the shape of a laser beam or to generate multi-spot patterns^20–24^ (see Fig. 1c).

A characteristic property that distinguishes AODs from other light modulating devices is that the motion of the acoustic wave naturally introduces a time variant light modulation which effectively introduces a degree of incoherence in the diffracted light. This effect has been applied e.g. for optical frequency shifting, to generate optical frequency combs for heterodyning applications^25,26,^ and to focus a laser beam through scattering media^27^. However, to the best of our knowledge, an artifact-free holographic light modulation system based on this effect has not been investigated so far, despite image projection systems incorporating acousto-optic deflectors were already commercially deployed almost a century ago. A very early image projection system based on acousto-optic devices was the Scophony television system, which was developed in the 1930s^28,29^. It utilized an antique acousto-optic water cell, which was driven with a video signal to diffract moving line segments of a television image on a screen. This system was sophisticated and rather complicated because the lines of the television image projected on the screen moved in the same way as the acoustic wave in the AOD did. For this reason, an additional scanning device was required to stabilize the image on the screen. This Scophony system also provided the basis for the first real-time three-dimensional holographic video display based on acousto-optic modulation, developed by S. Benton's Spatial Imaging Group at MIT in the early nineties of the last century. The Mark II system in particular was able to compute synthetic fringe patterns and display them on a TeO_2_ acousto-optic cell, projecting at video rates aerial color images with horizontal parallax^30,31^. It is nevertheless an earlier development by Korpel et al. in 1969 that is more closely connected to our work. They demonstrated a simplified acousto-optic laser projection system which was able to form steady image lines with an acousto-optic deflector^32^. They modulated the television signal in such a way that the line elements (image points) were represented as frequency components in the AOD driving signal. Then, through an optical Fourier transform of the spatial frequencies displayed on the AOD, a static image line was formed on a screen. The motion of the acoustic wave merely introduces a time varying phase in the image after the Fourier transform, which does not affect the perceived intensity. This property is also known as the shift theorem of the Fourier transform^33^.

**Figure 2:**
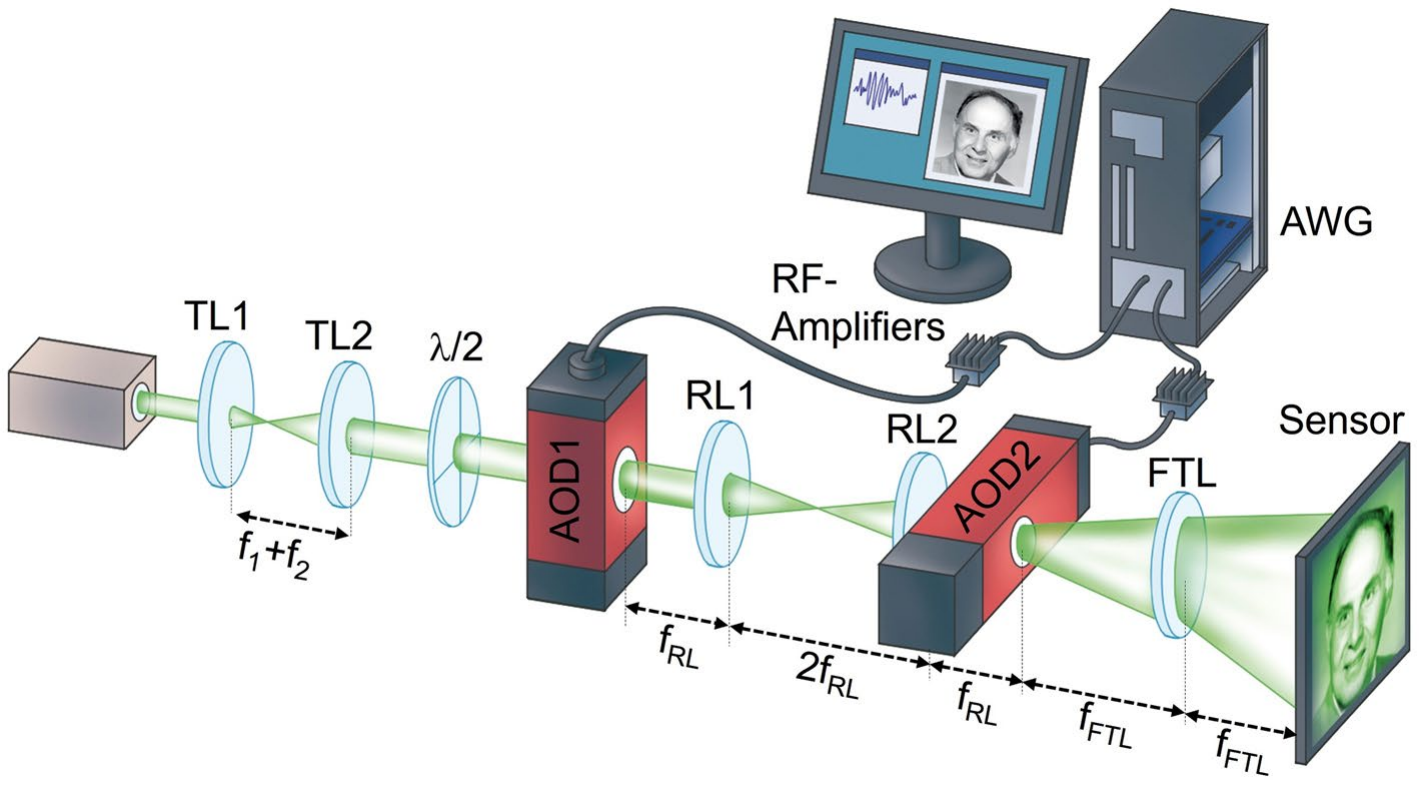
Conceptual setup of a holographic acousto-optic light modulation (HALM) system. A laser beam is successively modulated by a first acousto-optic deflector that displays a computer-generated hologram, and a second AOD that modulates the laser beam with a linear phase. The amplitude and phase of the hologram are computed from a digital target pattern and are encoded in the electric driving signal that controls the AOD. A lens performs an optical Fourier transform of the holographically modulated beam and reconstructs the one-dimensional target pattern that corresponds to the displayed hologram as a line in its focal plane. The position of the line is controlled through the linear phase displayed on the second AOD. The HALM system reconstructs two-dimensional patterns by sequentially reconstructing several lines at different positions.

In this work, we demonstrate theoretically and experimentally that this effect can be exploited in a holographic light modulation system to eliminate coherent artifacts entirely, producing images of very high fidelity. The concept of the proposed system is shown in Fig. 2. It utilizes two AODs that are situated in conjugated planes of a telecentric relay. The first AOD is driven with complexly modulated driving signals to display digital Fourier holograms, thereby acting as holographic SLM that eventually generates a one-dimensional target pattern in the back focal plane of a Fourier transform lens (FTL). The second AOD is driven with sinusoidal signals to act as a common deflector that controls the position of the reconstructed line. By synchronized holographic diffraction and deflection, any one or two-dimensional patterns can be formed by this system. The driving signals are synthesized with an arbitrary wavefront generator and amplified to achieve an optimal modulation depth of the AODs and thus optical efficiency. We demonstrate the capabilities of this so constituted holographic acousto-optic light modulation (HALM) system, with the example of a laser video projector that holographically reconstructs arbitrary intensity patterns without coherent artifacts.

In the 30's, acousto-optic projection pictures were considered the biggest and brightest of its time. Similarly to other venerable television technologies that found application in other imaging fields many decades after their invention, such as the Nipkow disk in laser microscopy, we expect that acousto-optic image display, in this modern incarnation as an artifact-free holographic laser pattern generator, finally finds the technical and commercial success that it was on the verge to obtain almost one century ago.

## Results

### Mathematical model and simulated reconstructions

A unique property of acousto-optic holograms displayed on an AOD is their continuous motion due to the propagation of the acoustic wave in the transparent AOD crystal. This causes temporal variations of the reconstructed intensity pattern which result in a reduction of coherent artifacts through an averaging effect. In the following section, we present the mathematical description of this effect and derive the required conditions under which complete artifact elimination takes place.

Acousto-optic holograms are displayed on an AOD by encoding a computer-generated hologram (CGH) *G*_*k*_ into the electrical driving signal. The CGH is calculated using the Gerchberg-Saxton algorithm^34^ with the input target pattern *i*_*n*_, and its amplitude and phase modulate the driving signal as explained in the methods section. The piezo-electric transducer forms this electrical signal into an acoustic wave, which in turn excites the acousto-optic hologram *G*(*x*,*t*) in the AOD crystal that diffracts the incoming light according to the amplitude and phase of the encoded CGH. The hologram *G*(*x*,*t*) matches the size *L* of the AOD aperture and is given by (see supplementary information S1):

1 $$G(x,t) = \left( {\left[ {\left( {\sum\limits_{k = 1}^{{N_{p} }} {G_{k} } \cdot \delta (x - kd_{P} + vt)} \right)*{\text{rect}}(x/d_{p} )} \right]*{\text{comb}}_{L} (x)} \right) \cdot {\text{rect}}(x/L).$$

The hologram propagates with the speed of the acoustic wave *v* and has a sample spacing given by *d*_*P*_ and a number of *N*_P_ pixels. The *rect* and *comb* functions are defined as in reference^33^. The hologram modulates the input wavefront *U*_in_ of the laser beam, and a lens performs an optical Fourier transform of the modulated beam to reconstruct the target pattern. The complex amplitude *U*’ of the reconstructed wavefront is given by (see supplementary information S2):

2 $$U^{\prime}(x^{\prime},t) = \left[ {\sum\limits_{n = - \infty }^{ + \infty } {i_{n} \delta (x^{\prime} - nd_{I} )} {\text{sinc}}\left( {\frac{{n\pi d_{p} }}{L}} \right){\text{exp}}\left( {i\frac{2\pi vtn}{L}} \right)} \right] * {\text{PSF}}(x^{\prime})$$

where

3 $${\text{PSF}}(x^{\prime}) = \frac{1}{{\sqrt {\lambda f_{L} } }}{\text{sinc}}\left( {\frac{{\pi x^{\prime}}}{{d_{I} }}} \right) * {\text{OFT}}\{ U_{in} \} .$$

The expression is composed of a periodically extended target pattern amplitude *i*_n_ (*i*_n_ = *i*_n+*N*p_), which multiplied by the sum of delta functions creates a grating of discrete image points with a spacing of:

4 $$d_{I} = \frac{{\lambda f_{L} }}{L}.$$

This grating is multiplied with a *sinc* function as consequence of the discretization of the hologram. In practice, the chosen sample rate of the hologram is very high because AODs are analogue (non-pixelated) modulators, so that these discretization effects are neglectable. The linear phase function in eq. (2) is the result of the motion of the hologram in the Fourier plane. The rectangular aperture with length *L* and the input wavefront define the shape of the PSF, which is responsible for the superposition of neighboring image points and the resulting coherent artifacts. The only time dependent factor in this expression is the linear phase. It has, however, a great effect on the outcome of the pattern, because it constantly changes the phase difference between image points and likewise the form of their interference pattern. Now it is assumed that the hologram is illuminated by a continuous wave laser, or by a pulsed laser with a pulse repetition rate that is much higher than the hologram switching rate of the AODs. Then, the fluence observed by a detector is the integral of all different intensity patterns formed during the integration time τ:

5 $$H(x^{\prime},\tau ) = \int_{{t_{0} }}^{{t_{0} + \tau }} {\sum\limits_{m = - \infty }^{\infty } {\sum\limits_{n = - \infty }^{\infty } {I_{m,n} } } } (x^{\prime}){\text{exp}}\left( {i\frac{2\pi vt(m - n)}{L} + i\theta_{m,n} (x^{\prime})} \right)dt$$

with

6 $$I_{m,n} (x^{\prime}) = \left| {{\text{PSF}}(x^{\prime} - md_{I} ){\text{PSF}}(x^{\prime} - nd_{I} )^{*} i_{m}^{{}} i_{n}^{*} {\text{sinc}}\left( {\frac{{m\pi d_{p} }}{L}} \right){\text{sinc}}\left( {\frac{{n\pi d_{p} }}{L}} \right)} \right|,$$

and

7 $$\theta_{m,n} (x^{\prime}) = {\text{arg}}\left\{ {I_{m,n} (x^{\prime})} \right\}.$$

The parameter *t*_0_ is an arbitrary point of time where the integration of the pattern by the detector starts. It is convenient to separate the expression in Eq. (5) into a time-independent sum *H*_P_ for which holds *m* = *n*, and the remaining sum *H*_S_ for which *m ≠ n* is true:

8 $$H(x^{\prime},\tau ) = H_{P} (x^{\prime},\tau ) + H_{S} (x^{\prime},\tau )$$

with

9 $$H_{P} (x^{\prime},\tau ) = \tau \sum\limits_{m = - \infty }^{\infty } ||{i_{m} {\text{PSF}}(x^{\prime} - md_{I} )}|| ,$$

and

10 $$H_{S} (x^{\prime},\tau ) = \int_{{t_{0} }}^{{t_{0} + \tau }} {\sum\limits_{m = - \infty }^{\infty } {\sum\limits_{n = m + 1}^{\infty } {I_{m,n} } } } (x^{\prime})2{\text{cos}}\left( {\frac{2\pi vt(m - n)}{L} + \theta_{m,n} (x^{\prime})} \right)dt.$$

The first sum *H*_P_ is equal to an incoherent superposition of the target image points and is hence an ideal optical reconstruction. The second sum *H*_S_ consequently summarizes all undesired contributions which come from the coherent superposition of image points, including holographic speckle. In this form, it is easy to see that *H*_S_ is zero if the integral over the cosine function is zero for all possible combinations of *m* and *n*. That implies that the integration time τ needs to be an integer multiple of the cosine period for all possible combination of *m* and *n*. This is the case when:

11 $$\tau = \frac{L}{v} = T_{{\text{T}}} .$$

This time is equal to the characteristic AOD response time *T*_T_, which is the duration that a hologram propagating at the speed of the acoustic wave *v* requires to pass the AOD aperture of length *L*. During the response time, the AOD performs a complete circular shift of the displayed hologram *G*, because the driving signal is periodic with the response time, i.e. the hologram matches the size *L* of the aperture. Therefore, *T*_T_ is also denoted as hologram cycle time in the further discussion.

**Figure 3:**
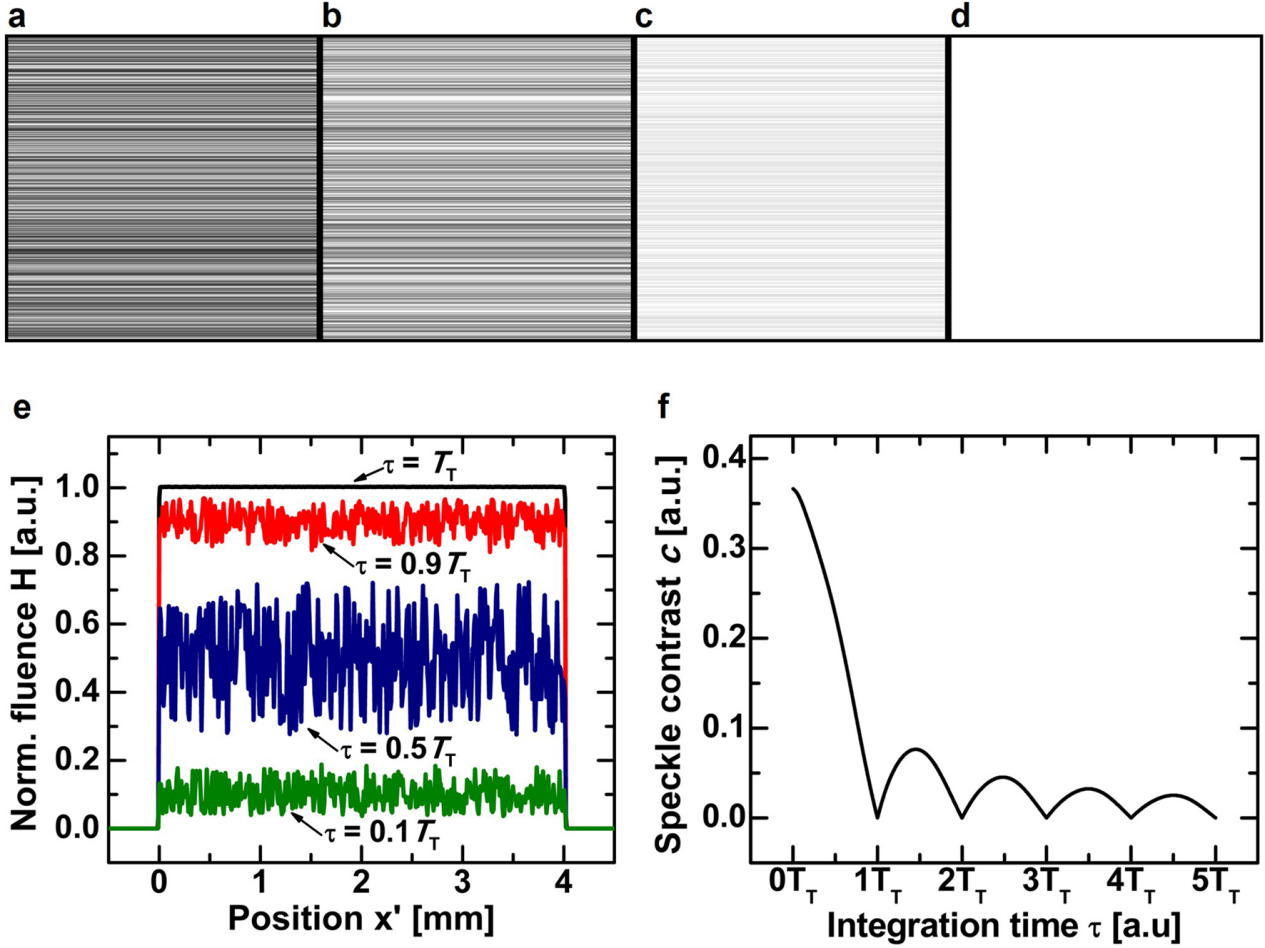
(**a**–**d**) Simulated reconstruction of a one-dimensional top-hat profile through an AOD for different detector integration times. (**a**) *τ* = 0.1*T*_T_; (**b**) *τ* = 0.5*T*_T_; (**c**) *τ* = 0.9*T*_T_; (**d**) *τ* = 1*T*_T_. The interference of neighboring image points results in holographic speckle, which is visible as high frequency noise in the observed pattern fluence. The motion of the hologram displayed on the AOD results in a time variant speckle pattern, which when integrated over the characteristic hologram response time *T*_T_, averages to zero. (**e**) Cross-section of the simulated top-hat profiles. (**f**) Speckle contrast *c* of the simulated top-hat profile as function of the detector integration time. The speckle contrast is zero when the integration time is an integer multiple of the response time *T*_T_. For longer acquisitions, the contrast reduces step-wise because the fluence of the speckle pattern oscillates while the fluence of the target pattern increases proportional with time.

Figure 3a–d show the simulated reconstruction of a one-dimensional top-hat profile through an AOD for different detector integration times τ. The vertically aligned reconstructions are extended in the horizontal dimension for better visibility. Figure 3e shows the corresponding cross-sections of the reconstructed patterns. We see that the patterns suffer from degradations for small integration times. Over time, these degradations reduce until a perfect top-hat profile is reconstructed by integrating over one complete hologram cycle. Hence, the reconstruction is free of coherent artifacts if the integration time is a multiple of *T*_T_. A common quantitative measure for image degradations resulting from coherent artifacts is the speckle contrast *c*, which is defined by:

12 $$c = \frac{\sigma }{{\hat{I}}}$$

where σ is the standard deviation of the pattern fluence and *Î* is its average value. Figure 3f shows the speckle contrast of the simulated top-hat profiles as a function of the integration time over a period of five hologram cycles. The speckle contrast behaves like a damped oscillator, because the fluence of the target pattern *H*_P_ increases proportional to the integration time, while the amplitude of the coherent sum *H*_S_ oscillates periodically with *T*_T_. After four hologram cycles, the speckle contrast stays below *c* = 0.02. Therefore, it is not required to precisely control τ for applications that allow integration times of several tenths of microseconds or even milliseconds. In high-speed applications, on the other hand, it is advantageous to match the integration time to a multiple of the response time to obtain a minimum speckle contrast while maintaining high pattern reconstruction rates. The response times of common AODs take values of a few microseconds, so that hundred thousands of artifact-free patterns can be formed per second.

The fact that AODs are capable of holographically reconstructing patterns that are equal to an incoherent reconstruction is an advantage towards conventional holographic modulators, which, in the best case, reconstruct a coherent superposition of image points with equal optical phases, what still results in undesired coherent contributions that reduce the resolution and fidelity of the reconstructed image^10^. Moreover, this averaging effect is an inherent mechanism of acousto-optic light modulation and has no drawbacks that reduce the reconstruction rate or light efficiency unlike common artifact correction methods.

As a side note, separable patterns, which are described by the product of one-dimensional functions, can be reconstructed by using two cascaded AODs as holographic modulators. This, however, leads to coherent artifacts which are not averaged out by the hologram motion. The discussion of this case is presented in the supplementary information (S3) of this work.

### Experimental reconstructions

The HALM system can reconstruct any two-dimensional intensity pattern by applying one AOD as holographic modulator to reconstruct image lines, and another AOD as line deflector. There are various applications that make use of laser intensity patterns, and each has different requirements as to the size, form, quality and reconstruction rate of the pattern. Natural images are perhaps the most challenging patterns to reconstruct, because they have a continuous intensity profile which requires many gray levels, and the closely situated image pixels interfere and produce substantial holographic speckle. The HALM system meets these requirements with an inherent reduction of coherent artifacts and an excellent 15-bit grayscale, which corresponds to the 15-bit voltage discretization of the driving signal. Moreover, AODs are non-pixelated modulators so that the pixel spacing can be chosen practically arbitrarily. Therefore, the common discretization effects of pixelated SLMs such as replicas and amplitude envelopes are avoided. The resolution of the reconstructed patterns is given by the time-bandwidth product of the AOD, which amounts to 461 diffraction limited spots in our configuration.

**Figure 4:**
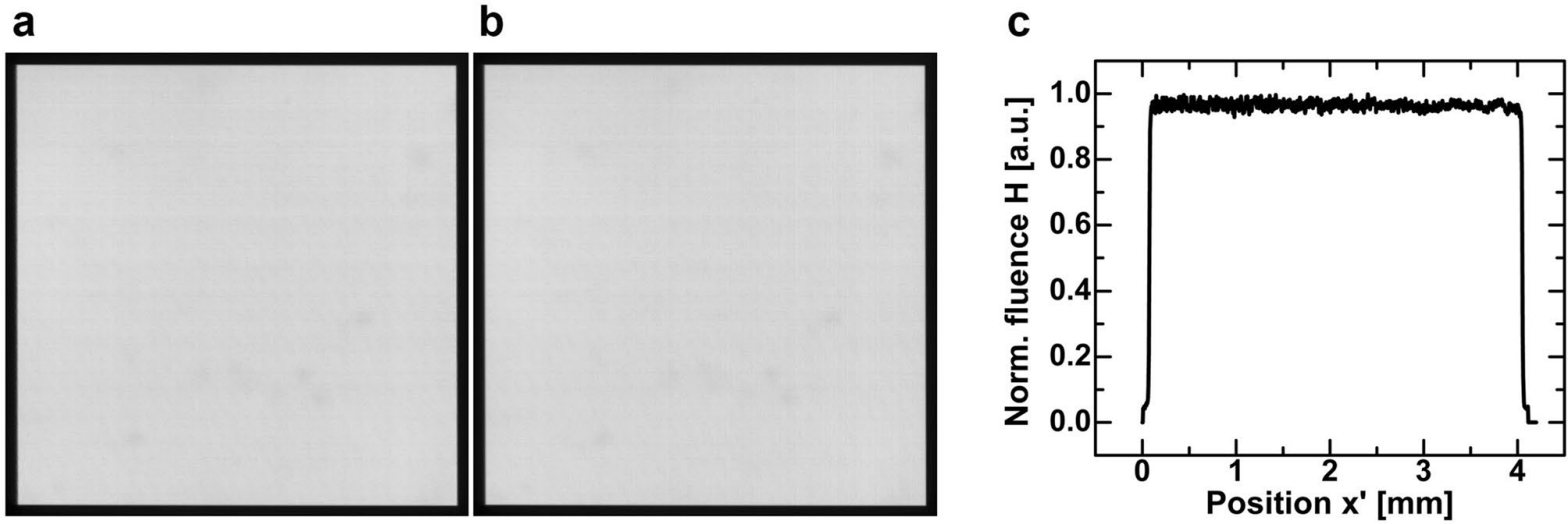
Experimentally reconstructed top-hat patterns. The patterns were recorded with detector integration times of (**a**) τ = 1*T*_T_ and (**b**) τ = 40*T*_T_. A cross-section of the pattern in (**a**) is presented in (**c**). Both patterns appear uniform and do not show visible degradations related to coherent artifacts. The similarity of the patterns, although captured with different integration times, supports the theoretical result which states that a pattern recorded for a multiple of the hologram cycle time (*T*_T_) is free of coherent artifacts.

Figure 4a,b show experimentally reconstructed top-hat patterns with a size of 300 × 300 image points, which were reconstructed with a 532 nm continuous wave laser. The graph in Fig. 4c shows the vertical cross-section of the image in Fig. 4a. The patterns were formed line-wise by holographically reconstructing 300 vertical top-hat profiles with the first AOD, and by deflecting the lines horizontally by the second AOD to form the two-dimensional pattern. The integration time for each line was controlled through the display time of the corresponding hologram, and was τ = *T*_T_ (9.23 μs) in Fig. 4a, and τ = 40*T*_T_ for each line in Fig. 4b, resulting in a total reconstruction time of 2.8 ms and 111 ms, respectively. The reconstructed patterns are virtually identical and do not show noticeable holographic speckle. The speckle contrast is *c* = 0.018 in both cases. This indicates that coherent artifacts are reliably eliminated, and that the integration over a single hologram cycle is sufficient to obtain an optimal result. A speckle contrast of zero was not expected because the pixel-pitch of the camera (4.65 μm) is smaller than the image pixel spacing (13.3 μm), so that the non-uniform PSF amplitude was sampled by about 3 camera pixels, what results in a non-zero speckle contrast even if no coherent artifacts are present. Also, experimental factors such as dust particles on the camera sensor, electronic noise and the frequency dependent behavior of the signal generator and the AODs affect the uniformity of the pattern.

The used AODs have a response time of *T*_T_ = 9.23 μs, resulting in a maximum line reconstruction rate of 108 kHz (as in Fig. 4a). Nevertheless, one must consider that when different holograms are displayed successively, one hologram cycle time is required to change the displayed hologram in the AOD aperture, and another cycle is necessary to eliminate coherent artifacts. Therefore, the effective maximum reconstruction rate is 54 kHz for arbitrary patterns. During a hologram transition, the modulation of the AOD is a mixture of the current and the subsequent hologram. That results in uncontrolled diffraction during the transition what introduces artifacts in the reconstructed pattern, which are here referred to as crosstalk noise (see supplementary information S4). The images in Fig. 4a,b do not show this noise related to hologram transition, because the image lines were identical and likewise their holograms. That, however, behaves differently for arbitrary patterns with distinct image lines.

**Figure 5:**
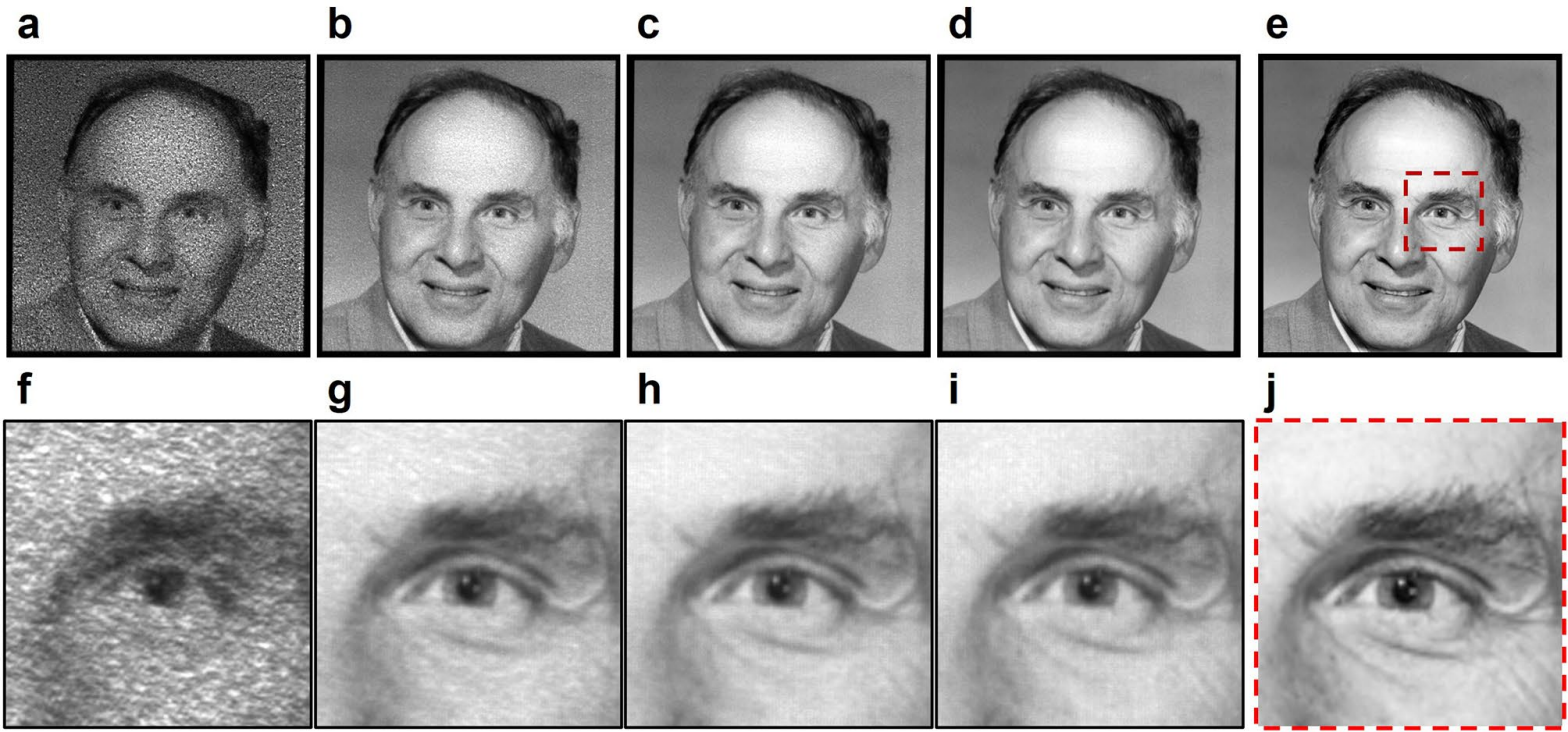
Experimentally reconstructed pattern “Arthur” (Reused with the permission of Nokia Corporation and AT&T Archives). (**a**–**d**) Line-wise reconstructed patterns (340 × 340 pixels) with different line integration times: (**a)** 1*T*_T_; (**b**) 5*T*_T_; (**c**) 10*T*_T_; (**d**) 20*T*_T_. (**f**–**i**) Zoomed excerpt of the corresponding image above. (**e**) Digital target pattern (340 × 340 pixels) and (**j**) zoomed excerpt (85 × 85 pixels). Coherent artifacts are effectively eliminated by the hologram motion. Nevertheless, the transition between different holograms displayed on an AOD introduces crosstalk noise. The visibility of the noise reduces rapidly with an increasing detector integration time τ.

Figure 5 shows the experimental reconstruction of a very detailed target pattern (a portrait of the 2018 Nobel laureate in Physics, Arthur Ashkin). Figure 5a–d show several reconstructions obtained with varying line integration times τ, and Fig. 5e shows the digital original for comparison. In Fig. 5f–j, zoomed excerpts of the corresponding images above are shown for a detailed comparison.

In the figure, from left to right, the detector integration time τ increases. Figure 5a was reconstructed with the smallest line integration time (τ = *T*_T_) and suffers from visible degradations, which were not observed for the top-hat profile. That suggests that these degradations are not coherent artifacts but crosstalk noise, which appears when different holograms are successively displayed on an AOD, and a temporally varying mixture of the holograms modulates the beam during the hologram transition resulting in uncontrolled diffraction.

Yet, we see that by increasing τ, the image quality is improved significantly until hardly any artifacts are visible. Longer line integration times reduce the contribution of the crosstalk noise because the fluence of the reconstructed line pattern increases proportional to τ, while the crosstalk noise is constant. That consequently results in an increased signal-to-noise ratio.

In the reconstruction with the best perceived image quality shown in Fig. 5d, even the finest details are visible, and the pixelated parts in the original image appear naturally smoothed by the PSF of the reconstructed points. Although experimentally captured by a camera (the resolution of the camera is about three times higher than the optical resolution limit), the images in Fig. 5c,d can compare to the digital original in Fig. 5e, demonstrating the excellent reconstruction quality that is achieved with the HALM system in practice. It is also to note that there is no zero-order visible in the image, because the diffracted and undiffracted light exit the AODs at different angles, resulting in a spatial separation of the zero-order. In LC-SLMs, the undiffracted light is not inherently separated from the diffracted light and typically forms an undesired bright spot in the center of the reconstructed pattern^35^.

The astonishing image fidelity that we obtain without additional speckle reduction methods indicates that the time-variant image phase, introduced by the hologram motion, reliably eliminates coherent artifacts for one-dimensional patterns, as predicted by theory. Although the reduction of crosstalk noise requires line integration times which are longer than one hologram cycle (the minimum for artifact-free reconstruction), our implementation of the HALM system is fast enough to achieve video rate holographic reconstructions.

**Figure 6:**
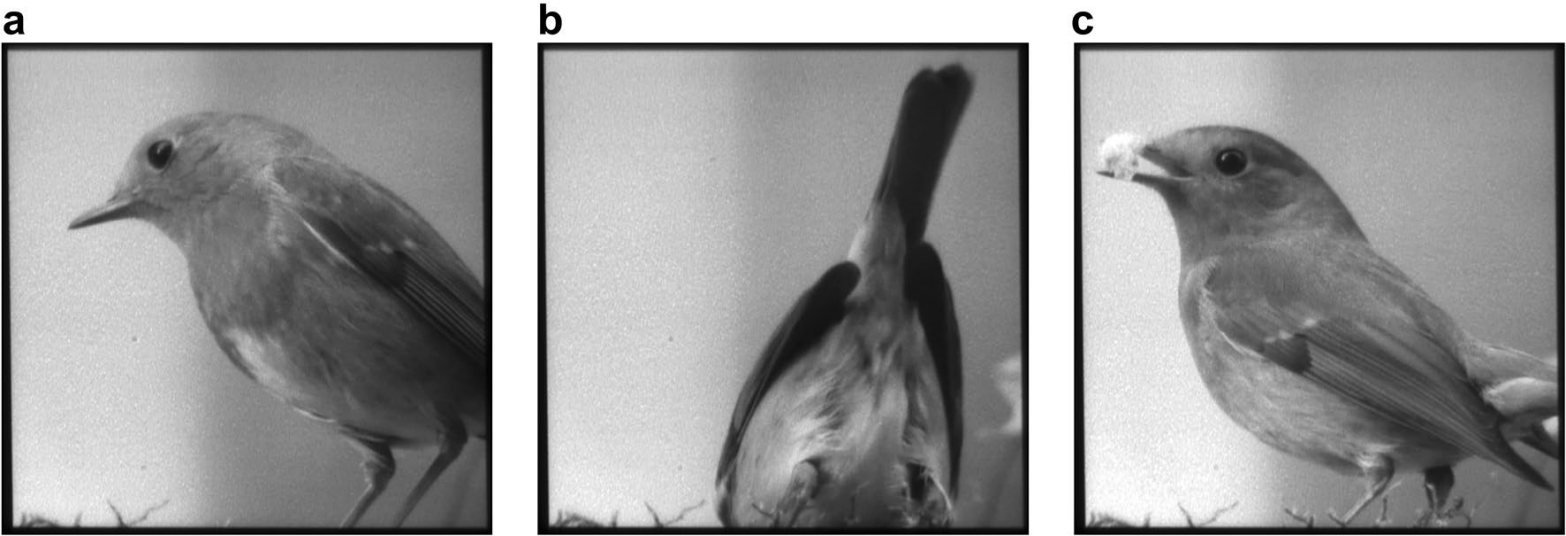
Frames of an experimentally reconstructed video (350 × 350 pixels). (**a**–**c**) The lively scene was reconstructed at 39 Hz with a line integration time of τ = 8T_T_ and demonstrates the capability of the HALM system for laser video projection. The video was recorded with different detector integration times and frame rates (see visualizations 1–5 in the supplementary material).

Figure 6a–c show three frames of an experimentally reconstructed video with a frame rate of 39 Hz (*τ* = 8*T*_*T*_), which is sufficiently fast to be perceived without flicker by the human eye. For comparison, a point-wise reconstruction of this video, performed by using both AODs in the deflector mode, would result in a maximum frame rate of only 1 Hz. The entire recorded video and additional reconstructions with *τ* = 1, 5, 10 and 20*T*_T_ are provided in the supplementary material of this work (Visualizations 1–5). According to the size of the image (*N*_L_ = 350 lines), we obtained frame rates of about 310 Hz, 62 Hz, 31 Hz and 15 Hz for those videos, respectively.

The combined light efficiency of both AODs is about 50% or more across their bandwidth. The average diffraction efficiency of the calculated holograms is 90%. Therefore, the average light efficiency for a reconstructed image line amounts to 45% in average. That is less than the efficiency of LC-SLMs, which typically achieve efficiencies of above 95%, but it is a reasonable trade-off for virtually perfect reconstructions. Furthermore, AODs typically have a large optical damage threshold (> 500 W/cm^2^) which enables them to handle higher input powers than LC-SLMs (~ 5 W/cm^2^), so that they effectively allow to output substantially larger optical powers despite their lower light efficiency.

## Discussion

In this work, we demonstrated theoretically and experimentally that the motion of holograms, displayed on an acousto-optic deflector, inherently eliminates coherent artifacts in holographically reconstructed laser patterns. That means no additional correction methods are required, and related impairments of the modulation performance are avoided. Based on this phenomenon, we set up a system for holographic acousto-optic light modulation (HALM) and demonstrated its capability as arbitrary laser pattern generator.

In the absence of coherent artifacts, we obtained holographically reconstructed patterns of astonishing quality. Here also came into play the analogue modulation of AODs, which avoids pixelation effects and enables a continuous modulation depth that is only restricted by the voltage discretization of the driving signals (15bit). Furthermore, we benefited from the inherent spatial separation of diffracted and undiffracted light through the AODs. Also the full-complex modulation capability of AODs contributed to superior image qualities in comparison to phase-only SLMs, because degradations associated with the omitted amplitude modulation of phase-only holograms are avoided^36^. This formidable pattern shaping capability, combined with the high optical power threshold of AODs, suggests that it may serve in applications with an elevated need for accurate and high intensity gray-scale patterns, such as in large venue laser projectors, laser material processing and additive manufacturing.

The particular implementation of the HALM system studied in this work is capable of reconstructing 54 thousand lines per second, owing to the fact that one hologram cycle of 9.23 µs is required to change a displayed hologram, and at least one more cycle is required to obtain an artifact-free reconstruction. To make use of the maximum reconstruction rate in future works, we will investigate to implement additional optical hardware in the system to filter the crosstalk noise that arises during hologram transitions on the AODs.

Nevertheless, we achieved an appealing image quality for reconstruction rates of more than 13 thousand lines per second. That amounted to 39 Hz for a video with an image size of 350 lines and is fast enough to enable applications where the projected pattern is perceived by the human eye, such as laser light shows, cinematic projections or laser-based illumination systems. The maximum resolution supported by our HALM configuration was 461 pixels and could be increased in combination with large angle scanning systems^37^.

Nowadays, AODs are vastly applied in beam steering and scanning applications in all fields of optics and photonics. It might be of great interest to users of these systems, that HALM can be directly implemented into conventional acousto-optic deflector systems by incorporating a signal generator that can form complexly modulated driving signals. This simple modification allows to benefit from a greatly increased flexibility of their existing AOD systems and a higher performance in suitable applications, as was recently demonstrated in the field of optical micro-manipulation^38^.

In conclusion, the combination of the immense switching speed of AODs, their full holographic modulation capabilities and their inherent holographic speckle reduction mechanism provides for new applications in fields where holographic light modulation is used to form intensity patterns.

## Materials and methods

### Spatial light modulation with acousto-optic deflectors

Light modulation through acousto-optic deflectors (AODs) is based on the acousto-optic effect, which describes the perturbation of the refractive index in a medium due to an acoustic wave traveling in it. In an AOD, a piezo-electric transducer is driven by an electrical signal and launches acoustic waves in the attached transparent crystal that exhibits the acousto-optic effect. The resulting refractive index perturbation in the crystal acts as a diffraction grating for a transmitted light wavefront. In its common application as deflector, an AOD is driven by a sinusoidal driving signal with a constant frequency, which creates a phase grating in the crystal that modulates the transmitted beam approximately with a linear phase^33^, similar to a prism. The resulting deflection angle is proportional to the frequency of the signal. Commonly, the laser beam is incident at the Bragg angle on the AOD, which increases the diffraction efficiency for the first diffraction order and cancels out all other orders, due to interference in the thick index grating created by the sound wave. The range of frequencies where the Bragg condition is met with sufficient precision and thus, at which an AOD can perform an efficient deflection of a laser beam, is specified by its bandwidth. The center of the bandwidth is indicated by a central driving frequency *f*_C_.

More complicated driving signals with amplitude and phase modulations can generate complex gratings (holograms) in an AOD that tailor the transmitted laser beam into a one-dimensional diffraction pattern^33^. We control the properties of the holograms with the amplitude and phase modulation of a carrier sinusoidal driving signal *S*(t):

13 $$S(t) = A(t)\sin \left( {2\pi f_{C} t + \varphi (t)} \right).$$

The acoustic wave launched by the piezo-electric transducer in response to this modulated electrical driving signal propagates with speed *v* in the crystal and creates a spatiotemporal refractive index perturbation $\tilde{n}$ with the form:

14 $$\tilde{n}(\zeta ) = \gamma A(\zeta )\sin \left( {2\pi f_{C} \zeta + \varphi (\zeta )} \right),$$

15 $$\zeta = \frac{x}{v} + (t - \Delta t).$$

The time delay Δ*t* takes into account the electronic and acoustic propagation of the signal before it reaches the AOD aperture, and *γ* is a material-specific constant of the crystal. A light wavefront passing through this index-modulated crystal with thickness *d* experiences an effective spatial phase modulation *H* which is approximately given by^33^:

16 $$H(x,t) \approx \frac{\gamma d\pi }{{\lambda_{0} }}A(\zeta )\exp \left( {i\varphi (\zeta )} \right)\exp \left( {i\varphi_{L} (\zeta )} \right),$$

17 $$\varphi_{L} (\zeta ) = 2\pi f_{C} \zeta .$$

We see that the temporal amplitude and phase modulation encoded in the driving signal is directly transferred into an equivalent one-dimensional spatial amplitude and phase modulation of the transmitted light wavefront. We refer to the combined spatial amplitude and phase modulation which gives rise to a diffraction pattern as hologram *G* with:

18 $$G(\zeta ) = A(\zeta )\exp \left( {i\varphi (\zeta )} \right).$$

Ignoring the constant factors in Eq. (16) and expressing the modulation in terms of the hologram, we obtain:

19 $$H(x,t) \propto G(\zeta )\exp \left( {i\varphi_{L} (\zeta )} \right).$$

The linear phase ramp *ϕ*_L_ deflects the diffraction pattern reconstructed by *G* at a certain angle according to *f*_C_, which results in a spatial separation of the pattern and the undiffracted light. That means the modulation *H* of the AOD is equal to the hologram *G*. This simple relationship between the modulation of the driving signal and the resulting wavefront modulation allows to display holograms on AODs by directly coding them into the driving signal. We also see from Eq. (18) that we can modulate both phase and amplitude of a wavefront with AODs. This is an advantage compared to other candidates for holographic image projection such as LC-SLMs, because no phase-only or amplitude-only approximation of the hologram is required.

### Displaying holograms on AODs

**Figure 7:**
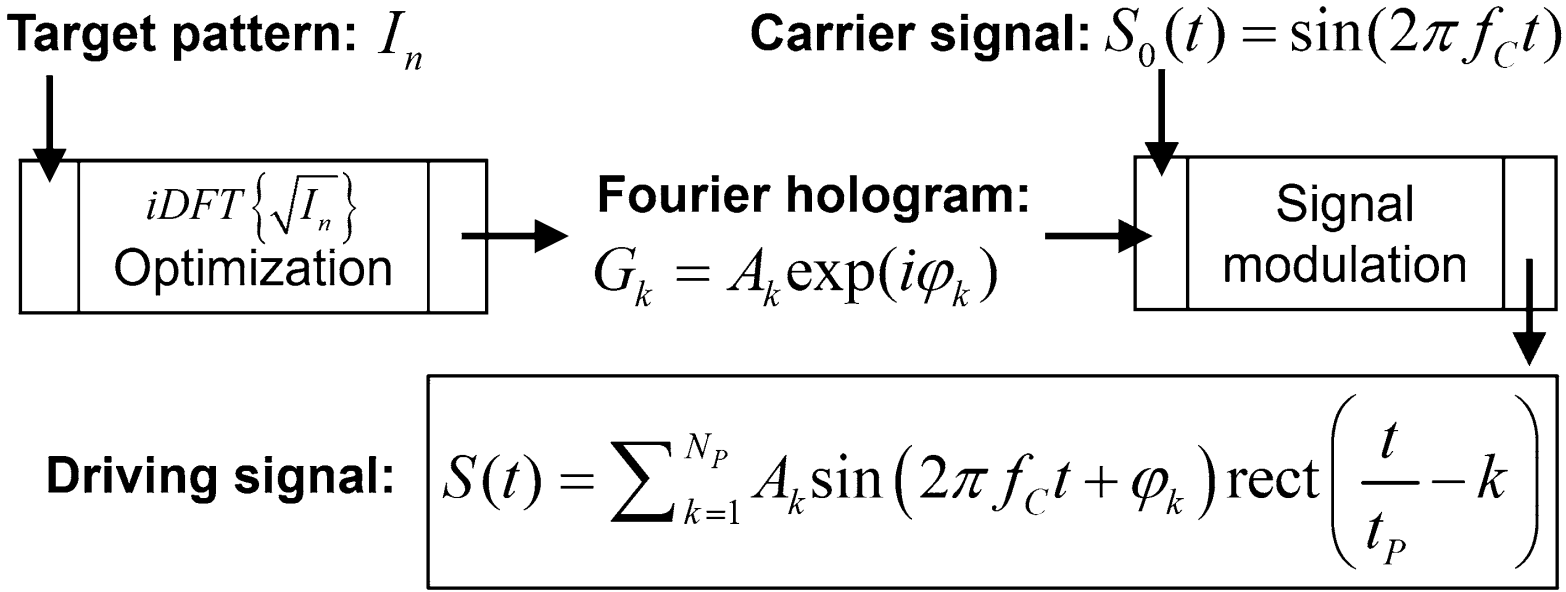
Calculation of the holographic driving signal. The Fourier hologram is calculated with an inverse Fourier transform of the target pattern *I*_n_. An iterative algorithm optimizes the hologram diffraction efficiency. The amplitude and phase of the hologram are encoded in the carrier signal, taking into account the size of the AOD aperture *L* and the number of hologram sample points *N*_P_.

The electronic driving signal controls the amplitude and phase of the hologram which is displayed on an AOD, and hence the generated intensity patterns. Since we focused on the reconstruction of one- and two-dimensional patterns, we utilized Fourier holograms which are fast to calculate and easy to reconstruct via an optical Fourier transform setup. The flowchart in Fig. 7 describes the calculation of an AOD driving signal based on a one-dimensional digital target pattern *I*_n_. The digital Fourier hologram *G*_k_, which reconstructs the target pattern when displayed on the AOD, is obtained by an inverse discrete Fourier transform (*iDFT*) of the square root of the pattern:

20 $$G_{k} = iDFT\left\{ {\sqrt {I_{n} } } \right\}(k) = A_{k} \exp (i\varphi_{k} ),{\text{ k}} \in \left[ {1,N_{P} } \right].$$

The amplitude and phase modulations *A*_k_ and *ϕ*_k_ that characterize the hologram are directly found in the Euler notation of the complex result of the *iDFT*. However, the diffraction efficiency of the Fourier holograms computed in this way is usually very low due to the amplitude modulations of the signal. We optimized this efficiency with a modified Gerchberg-Saxton algorithm^34,39^, which approximates a phase-only hologram (kinoform), to reconstruct a target pattern with improved transparency e.g., with an optimized efficiency. The improvement of the diffraction efficiency depends on each pattern individually, but typically shows a 100-fold increase.

The optimization loop had 1000 iterations, which was sufficient for convergence. There were no constraints on *A*_k_ or *ϕ*_k_. In principle, amplitude and phase discontinuities in the signal cause pixelation effects, similar to the effects observed when displaying holograms on pixelated LC-SLMs. To avoid that, we used high hologram sample rates by zero-padding the digital target pattern.

We used an office PC (CPU: i5-6400 @ 2.7 GHz, 8 Gb RAM, Intel HD Graphics 530) for the hologram calculation. The calculation was performed in MATLAB and took about 1 min for one image with 350 lines and 1000 iterations of the GS algorithm. We did not optimize the hologram calculation in this regard, and there is certainly a high potential to increase the calculation speed by performing the fft on a dedicated GPU and by using more potent computer hardware if required.

The holograms that reconstruct the different image lines have slightly different diffraction efficiencies, and the image lines also require different optical powers (since there are brighter and darker image lines). Both the target power and the hologram diffraction efficiency can be calculated prior to the experimental reconstruction. Accordingly, we added an additional amplitude modulation to the final holograms to account for these differences in hologram efficiency and target power.

The digital hologram *G*_k_ represents a sampled version of the physical hologram *G*(*x*,*t*), whose amplitude and phase is encoded in the driving signal according to Eq. (13). The hologram matches the size of the AOD aperture *L* and is discretized by *N*_P_ sample points. A physical hologram pixel has then the size *d*_P_:

21 $$d_{P} = \frac{L}{{N_{P} }}.$$

Consequently, the duration *t*_P_ of the driving signal that corresponds to each pixel is:

22 $$t_{P} = \frac{{d_{P} }}{v}.$$

The driving signal *S*(*t*) is hence a series of connected sinusoidal segments which carry the holographic modulation *A*_k_ and *ϕ*_k_ of each pixel:

23 $$S(t) = \sum\nolimits_{k = 1}^{{N_{P} }} {A_{k} \sin \left( {2\pi f_{C} t + \varphi_{k} } \right)rect\left( {\frac{t}{{t_{P} }} - k} \right)} .$$

**Figure 8:**
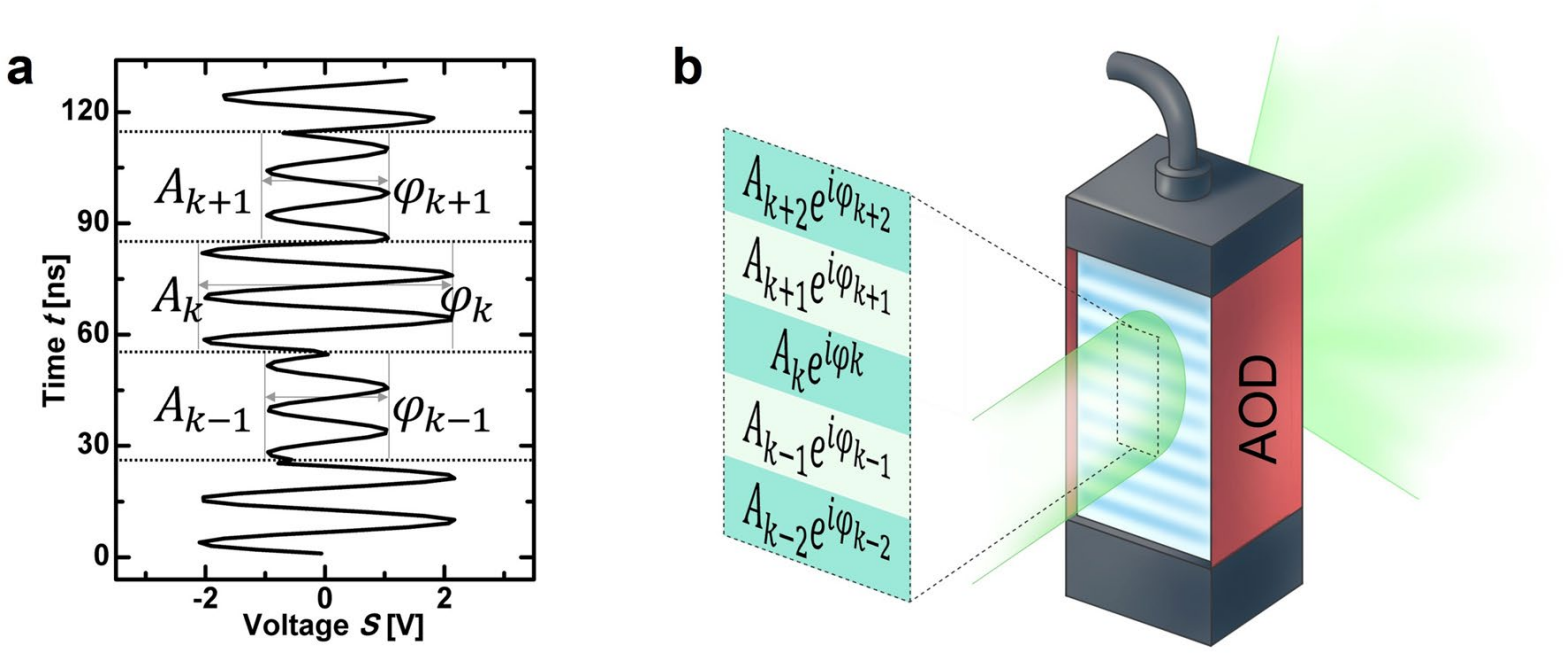
Electronic and optical representation of the digital hologram. (**a**) Electrical driving signal with encoded Fourier hologram *G*_k_. These complex modulated driving signals are generated with an arbitrary wavefront generator. (**b**) The reconstructed physical hologram *G*(x,t) displayed by the AOD has a similar amplitude and phase modulation.

The *rect* function generates the sinusoidal segments with duration *t*_P,_ which represent one hologram pixel each. The graph in Fig. 8a shows an exemplary driving signal that carries a discrete hologram, and Fig. 8b illustrates the resulting physical hologram on the AOD. The phase and amplitude of the different pixels are indicated. In our experiments, the frequency of the carrier signal was the central driving frequency of the AODs (*f*_C_ = 83.5 MHz) because the diffraction efficiency of the AODs is optimized around this frequency.

We generated the signal using an arbitrary wavefront generator (AWG) with a voltage discretization of 15 bits. That means the hologram amplitude and phase are also discretized with 15 bits (yielding more than 32 thousand amplitude and phase values), because both result in a change of the electronic signal amplitude.

### Experimental system

The HALM system employs two perpendicularly arranged AODs (AA Opto-Electronic DTSXY-400-488.561-010), with a central driving frequency of *f*_C_ = 83.5 MHz and a bandwidth of *B* = 50 MHz. The acoustic speed in the modulating TeO_2_ crystals is *v* = 650 m/s, and the aperture has a diameter of *L* = 6 mm. The laser beam, with wavelength *λ* = 532 nm (Changchun New Industries, MGL-III-532-200mW), enters the AODs at the Bragg angle to optimize the diffraction efficiency for the first diffraction order. A beam expander matches the output beam diameter of the laser (1.2 mm) to the aperture size of the AODs (6 mm), and the half-wave-plate rotates the beam polarization according to the orientation of the TeO_2_ crystals to optimize the diffraction efficiency. A telecentric relay conjugates the modulation planes of the AODs to account for their spatial separation. The AODs subsequently modulate the laser beam, and a lens with focal length *f*_L_ = 150 mm performs an optical Fourier transform of the modulated wavefront to reconstruct the images onto the sensor of a camera (QICAM 12bit Fast 1394).

The arbitrary wavefront generator (Spectrum M4i.6631-x8), with an output bandwidth of 400 MHz, generates the AOD driving signals with a 15-bit voltage discretization and an output sample rate of about 1.25 GS/s. Its maximum peak-to-peak output voltage is 4 V, and two amplifiers (Mini-Circuits ZHL-1010 +) increase the RF power applied to the AOD cells to achieve optimal modulation depth and thus optical efficiency.

A transition between holograms displayed on an AOD requires the time needed for the acoustic wave in the crystal to pass the aperture *L* of the AOD. Hence, the characteristic response time *T*_T_ is given by:

24 $$T_{T} = \frac{L}{v}.$$

This is the minimum time required for the AODs to switch between any one- or two-dimensional separable patterns. Consequently, the maximum frame rate of the AODs *r*_0_ is:

25 $$r_{0} = \frac{1}{{T_{T} }}.$$

For our setup, we obtain *T*_T_ = 9.23 μs and *r*_0_ = 108.33 kHz, meaning that more than 10^5^ different separable patterns can potentially be reconstructed in 1 s. When we reconstruct non-separable patterns, then this rate reduces inversely to the number of lines in the image. For an image that contains *N*_L_ lines, the maximum frame rate *r* is:

26 $$r = \frac{{r_{0} }}{{N_{L} }}.$$

The maximum size of the reconstructed patterns is given by the number of resolvable spots *N*_P_ that can be simultaneously reconstructed with an AOD. It is approximately given by the product of its bandwidth *B* and the response time *T*_T_^40^:

27 $$N_{P} = T_{T} B.$$

We obtain a value of *N*_P_ = 461 points for our setup (*B* = 50 MHz). Therefore, we can reconstruct up to 5 × 10^7^ points per second with one AOD. The variable parameter in the setup is the laser beam diameter *L*. A larger diameter increases the possible pattern size, and a smaller diameter increases the reconstruction speed (requiring less time for the sound wave to cross the illuminated area), so that the beam diameter serves as a flexible parameter in adapting the system properties for different applications.

### Correction of AOD specific image degradations

Besides coherent artifacts, we observed two image degrading effects which are specific to light modulation with AODs. One issue consists of degradations that come from undesired crosstalk formed during a transition from one hologram to another, due to the finite velocity of the sound wave. This effect is described in more detail in the supplementary information S4. In the experiment, it was reduced by repeating the holograms so as to increase the display time of the correct information versus the crosstalk period (i.e., an increased signal-to-noise ratio).

Another problem is the non-uniform diffraction efficiency of an AOD across its bandwidth, which results in an undesired amplitude modulation of the reconstructed pattern. We solved this problem by measuring the non-uniformity *r* and calculating a modulated target pattern *Î* given by:

28 $$\hat{I} = \frac{I}{r}.$$

The hologram calculated on the basis of *Î* reconstructs a pattern which neutralizes the non-uniformity and effectively reconstructs the target pattern *I*.

## Supplementary Information

Supplementary Information 1.

Supplementary Information 2.

Supplementary Information 3.

Supplementary Information 4.

Supplementary Information 5.

Supplementary Information 6.

Supplementary Information 7.
