# Supplementary material for: Artifact-free holographic light shaping through moving acousto-optic holograms
Source: Sci Rep. 2021 Oct 28;11:21261. doi: 10.1038/s41598-021-00332-4 (PMC8553788; doi:10.1038/s41598-021-00332-4)
Supplement: Supplementary file 1 — Supplementary Information 1. [file 41598_2021_332_MOESM1_ESM.docx]

Artifact-free holographic light shaping through moving acousto-optic holograms: Supplementary information

Dorian Treptow,^1,2,*^ Raúl Bola,^1,2^ Estela Martín-Badosa^1,2^ and Mario Montes-Usategui^1,2^

^1^Optical Trapping Lab – Grup de Biofotònica, Departament de Física Aplicada, Universitat de Barcelona

^2^Institut de Nanociència i Nanotecnologia (IN²UB), 08028 Barcelona, Spain

^*^Corresponding author: dorian.treptow@ub.edu

S1: Mathematical representation of a Fourier hologram displayed on an AOD

A complex modulated driving signal that carries a hologram (*G*_k_=*A*_k_exp(iϕ_k_)) is given by:

 .

This displays a hologram on the AOD which is given by:

 .

This can be rewritten to:

$G(x,t)=\sum_{k=1}^{N_{p}} G_{k}\text{rect}\left(\frac{vt+x}{d_{p}}-k\right)$ .

The signal is periodic with *L*=*vT*_T_ to perform a continuous modulation of the laser beam. Therefore, the hologram is convolved with a comb function with frequency *L*, and multiplied with a rect function which conserves the aperture of the AOD:

$G(x,t)=\left(\sum_{k=1}^{N_{p}} G_{k}\text{rect}\left(\frac{vt+x}{d_{p}}-k\right)*\text{comb}_{L}(x)\right)\cdot\text{rect}\left(\frac{x}{L}\right)$ .

The expression of eq.(1) in the manuscript is finally obtained by introducing:

$\text{rect}\left(\frac{vt+x}{d_{p}}-k\right)=\delta(x-kd_{p}+vt)*\text{rect}\left(\frac{x}{d_{p}}\right)$ .

S2: The reconstructed wavefront

The optical Fourier transform of the modulated beam wavefront is given by:

$U'(x',t)=FT\left\{U_{in}(x)G(x,t)\exp\left(i2\pi f_{C}\left(t+\frac{x}{v}\right)\right)\right\}$ .

First, the shape of the input wavefront, the aperture rect function and the scaling factor of the optical Fourier transform are summarized as PSF:

 .

The spatio-temporal linear phase in eq. results in a delta function which displaces the diffraction pattern according to the central driving frequency *f*_C_:

$\text{FT}\left\{\exp\left(i2\pi f_{C}\left(t+\frac{x}{v}\right)\right)\right\}=\delta\left(x'-\frac{\lambda f_{L}f_{C}}{v}\right)\exp\left(i2\pi f_{C}t\right)$.

The remaining terms of the hologram *G* and the temporal linear phase result in:

$$\begin{matrix} \text{FT}\left\{G(x,t)\right\}=\text{FT}\left\{\left[\left(\sum_{k=1}^{N_{p}} G_{k}\cdot\delta(x-kd_{p}+vt)\right)*\text{rect}(x/d_{p})\right]*\text{comb}_{L}(x)\right\} \\ =\left[\sum_{k=1}^{N_{p}} G_{k}exp\left(i\frac{2\pi kd_{p}x'}{\lambda f_{L}}\right)\right]\text{exp}\left(i\frac{2\pi vtx'}{\lambda f_{L}}\right)\text{sinc}\left(\frac{\pi x'd_{p}}{\lambda f_{L}}\right)\cdot\text{comb}_{\frac{1}{L}}\left(\frac{x'}{\lambda f_{L}}\right). \end{matrix}$$

The multiplication with the comb function discretizes eq. to:

 .

Here, the relationships *L*=*N*_P_*d*_P_ and *d*_I_=λ*f*_L_/*L* were applied. The term in the square brackets is equal to a discrete Fourier transform of the hologram *G*_k_ which results in the target pattern *i*_n_:

$i_{n}=\sum_{k=1}^{N_{p}} G_{k}\text{exp}\left(i\frac{2\pi kn}{N_{P}}\right)$ .

Introducing this result in eq. and adding all other terms from eq., we obtain for the complete expression of the reconstructed wavefront:

$U'(x',t)=\left[\sum_{n=-\infty}^{+\infty} i_{n}\delta(x'-nd_{I})\text{sinc}\left(\frac{\pi nd_{p}}{L}\right)\text{exp}\left(i\frac{2\pi vtn}{L}\right)\right]*\text{PSF}(x')$.

S3: Artifact suppression in two-dimensional pattern reconstruction

The successive modulation of a laser beam by two perpendicular aligned AODs allows to form separable two-dimensional patterns. The effective modulation of two AODs situated in conjugate planes of a telecentric relay is described by the multiplication of the holograms displayed on each AOD. Because of the separability, the resulting intensity pattern is the product of the two one-dimensional intensity patterns generated by each hologram:

$U'(x',y',t)=U'(x',t)U'(y',t)$.

The intensity of the two-dimensional pattern is given by:

$I(x',y',t)=U'(x',t)U'*(x',t)U'(y',t)U'*(y',t)$.

Introducing eq. in eq. yields:

$$I(x',y',t)=\sum_{m=-\infty}^{\infty} \sum_{n=-\infty}^{\infty} \sum_{r=-\infty}^{\infty} \sum_{s=-\infty}^{\infty} I_{\begin{matrix} m,r \\ n,s \end{matrix}}(x',y')\text{exp}\left(i\frac{2\pi vt(m+r-n-s)}{L}+i\theta_{\begin{matrix} m,r \\ n,s \end{matrix}}(x',y')\right)$$

where

$$\begin{matrix} I_{\begin{matrix} m,r \\ n,s \end{matrix}}(x',y')=\left|\text{PSF}(x'-md_{I},y'-rd_{I})\text{PSF}^{*}(x'-nd_{I},y'-sd_{I})i_{m}i_{n}^{*}i_{r}i_{s}^{*}\right| \\ \text{ }\times\left|\text{sinc}\left(\frac{m\pi d_{p}}{L}\right)\text{sinc}\left(\frac{n\pi d_{p}}{L}\right)\text{sinc}\left(\frac{r\pi d_{p}}{L}\right)\text{sinc}\left(\frac{s\pi d_{p}}{L}\right)\right|, \end{matrix}$$

and

$$\theta_{\begin{matrix} m,r \\ n,s \end{matrix}}(x',y')=\text{arg}\left\{I_{\begin{matrix} m,r \\ n,s \end{matrix}}(x',y')\right\}.$$

By integration over time we obtain for the two-dimensional fluence recorded by the detector:

$H(x',y',\tau)=\int_{t_{0}}^{t_{0}+\tau} \sum_{m=-\infty}^{\infty} \sum_{n=-\infty}^{\infty} \sum_{r=-\infty}^{\infty} \sum_{s=-\infty}^{\infty} I_{\begin{matrix} m,r \\ n,s \end{matrix}}(x',y')\text{exp}\left(i\frac{2\pi vt(m+r-n-s)}{L}+i\theta_{\begin{matrix} m,r \\ n,s \end{matrix}}(x',y')\right)dt$.

As in the one-dimensional case, all time-dependent summands (*m+r-n-s≠0*) are zero by integration over the response time *T*_T_. The time-independent part *H*_0_ (*m+r-n-s=0*) is given by:

$H_{0}(x',y',\tau)=\int_{t_{0}}^{t_{0}+\tau} \sum_{m=-\infty}^{\infty} \sum_{n=-\infty}^{\infty} \sum_{r=-\infty}^{\infty} I_{\begin{matrix} m,r \\ n,s \end{matrix}}(x',y')\text{exp}\left(i\theta_{\begin{matrix} m,r \\ n,s \end{matrix}}(x',y')\right)dt$,

where *s=m+r-n*. This sum can be further split into the incoherent sum of all images points *H*_P_, for which holds *m=n* respectively *s=r*, and the remaining undesired sum *H*_S_:

$H_{P}(x',y',\tau)=\tau\sum_{m=-\infty}^{\infty} \sum_{r=-\infty}^{\infty} \|i_{m,r}\text{PSF}(x'-md_{P},y'-rd_{P})\|$ ,

$H_{S}(x',y',\tau)=\int_{t_{0}}^{t_{0}+\tau} \sum_{m=-\infty}^{\infty} \sum_{n=m+1}^{\infty} \sum_{r=-\infty}^{\infty} I_{\begin{matrix} m,r \\ n,s \end{matrix}}(x',y')2\text{cos}\left(i\theta_{\begin{matrix} m,r \\ n,s \end{matrix}}(x',y')\right)dt$.

We see that besides the target pattern *H*_P_, also a part of the remaining sum persists after integration over *T*_T_. This suggests that the sets of image points addressed by the sum in eq. are coherent. Consequently, the two-dimensional reconstruction is not free of coherent artifacts. Even for longer integration times, *H*_S_ does not reduce because it is time independent.

This is illustrated in Figs. S1a-d, which shows the simulated reconstruction of a two-dimensional top hat profile for different integration times. Initially, a stripe pattern is observed for integration times much shorter than one hologram cycle as seen in Figs. S1a-b. Closer to the optimal integration time of *T*_T_, the pattern assumes the shape of a top hat profile, but visible degradations remain due to *H*_S_. The smooth features of *H*_S_ in Fig. S1d can be explained by a partial reduction of the coherent artifacts, which takes place because all summands for which holds *m+r-n-s ≠ 0* are zero when integrating over a multiple of *T*_T_. Figure S1e shows the behavior of the speckle contrast *c* of the simulated top hat profile as a function of the integration time. The contrast reduces rapidly with τ until a minimum is reached (*c*=0.077) by integrating over one hologram cycle. This value corresponds to the power of *H*_S_ and is obtained for all integration times that are an integer multiple of *T*_T_. For longer integration times than one hologram cycle, the speckle contrast oscillates but never falls below the minimum.


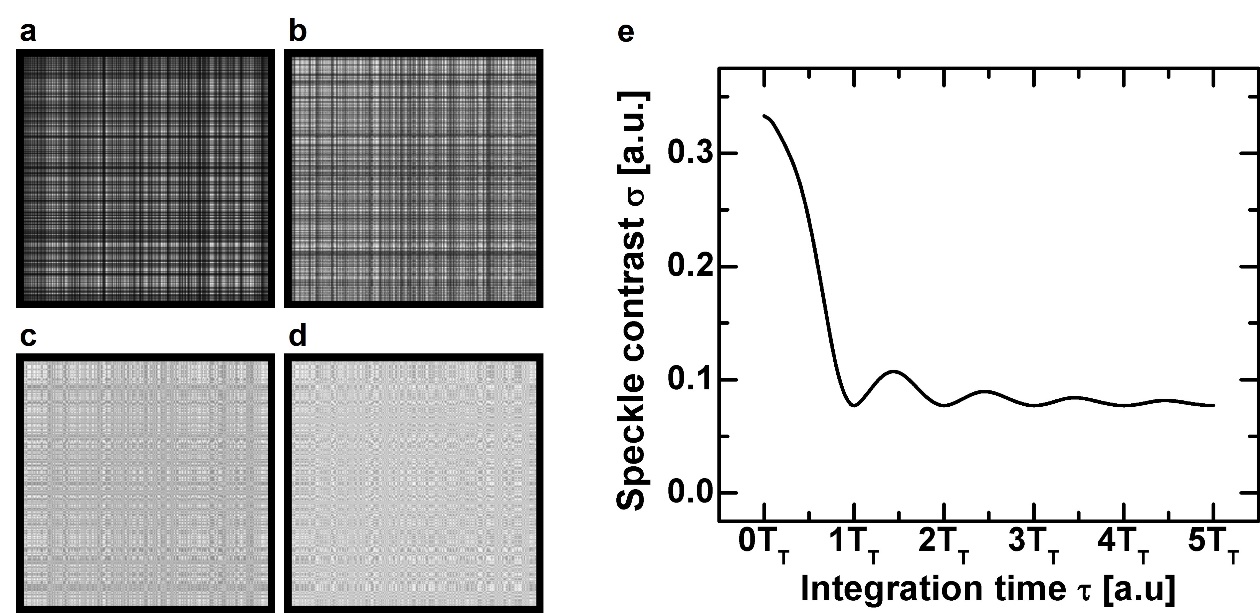


Fig. S1. (**a**-**d**) Simulated reconstruction of a top-hat profile with two perpendicular aligned AODs for different detector integration times; (**a**) τ=0.1T_T_; (**b**) τ=0.5T_T_; (**c**) τ=0.9T_T_; (**d**) τ=1T_T_. For short integration times, the reconstructed pattern suffers from strong degradations due to holographic speckle. The degradations reduce visibly when the integration time approaches an integer multiple of the AOD response time. (**e**) Speckle contrast c of the simulated top hat profile as function of the detector integration time. The minimum speckle contrast (c=0.077) is achieved by integrating the reconstructed image for an integer multiple of the AOD response time. The speckle pattern is reduced but not eliminated, because the two-dimensional modulation generates sets of coherent image points which form a time independent speckle pattern. Additional speckle reduction methods can reduce the contrast further.

Figures S2a-b show experimentally reconstructed two-dimensional top-hat patterns that were formed by displaying holograms corresponding to top-hat profiles on both AODs simultaneously. The detector integration times of τ=*T_T_* respectively τ=40*T_T_* here correspond to the total reconstruction times of 9.23μs and 0.37ms, which means that these patterns were reconstructed 300 times faster than in the one-dimensional case because no decomposition into lines was applied. However, these two-dimensional reconstructions suffer from coherent artifacts, whose characteristic appearance is in accordance with our simulation results. The speckle contrast is *c*=0.2 for both patterns.


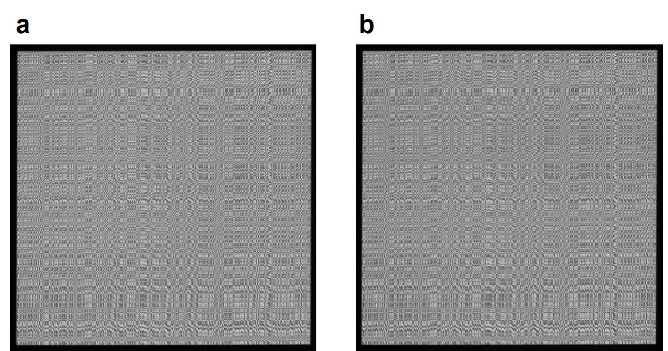


Fig. S2. Experimentally reconstructed top-hat patterns. The patterns were recorded with detector integration times of (**a**) τ=T_T_ and (**b**) τ=40T_T_, which corresponds to extremely high reconstruction rates of 108kHz and 2.7kHz respectively. We observe severe coherent artifacts that appear smooth and not granulated as common speckle noise. The previous theoretical results suggest that this comes from a partial elimination of the coherent artifacts through the hologram motion. The remaining degradations come from the interference of several sets of coherent image points.

S4: Hologram transition crosstalk noise

As any other type of SLM, also AODs require a certain amount of time to switch between different displayed holograms. In the case of AODs, this transition time *T*_T_ is the duration needed for the acoustic wave in the crystal to pass the modulating aperture of the AOD. During this time, the laser beam is modulated by the current and subsequent holograms, partwise, which introduces crosstalk noise.

Figures S3a-c illustrate the hologram transition in an AOD. The current hologram A and the subsequent hologram B move in x direction, almost perpendicular to the laser beam with diameter *L*. The first image (a) shows the modulation just before the transition (*t*=*T*_0_), when the beam is modulated by hologram A. Then, during the hologram transition (b, *T*_0_<*t*<*T*_0_+*T*_T_), the beam is partially modulated by hologram A and hologram B (crosstalk) in different proportions over time. That leads to degrading diffraction patterns during the transition. After the transition (c, *t*=*T*_0_+*T*_T_), the beam is only modulated by hologram B.

However, if the detector integration time is significantly longer than the transition time *T*_T_, then the crosstalk noise contributes little to the time averaged reconstructed intensity pattern. That means a sufficiently large number of repetitions of each hologram signal (i.e., increasing the display time of the holograms) can reduce the contribution of crosstalk noise.


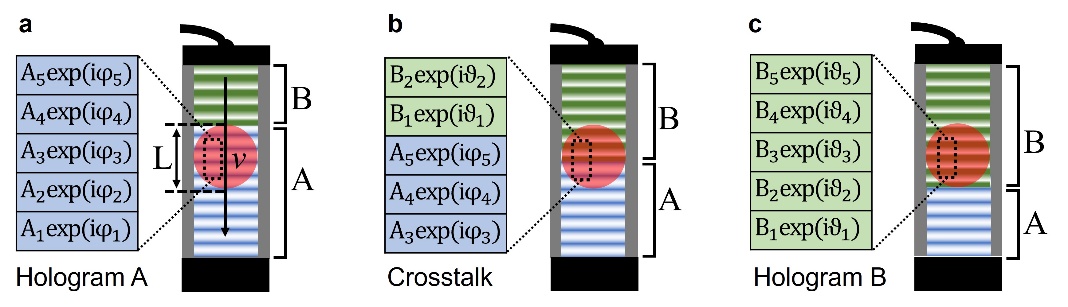


Fig. S3. Hologram transition in an AOD. (**a**) Hologram A is displayed by the AOD; (**b**) Holograms A and B are partially displayed during the transition, which leads to degrading crosstalk noise; (**c**) Hologram B is displayed.
