# Supplementary figures and images for: Artifact-free holographic light shaping through moving acousto-optic holograms
Source: Sci Rep. 2021 Oct 28;11:21261. doi: 10.1038/s41598-021-00332-4 (PMC8553788; doi:10.1038/s41598-021-00332-4)

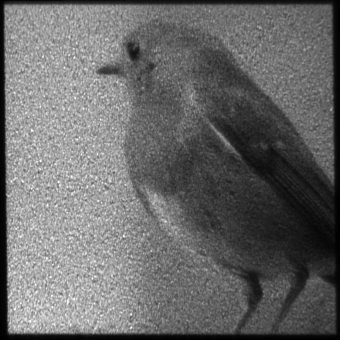

Supplement: Supplementary file 2 — Supplementary Information 2. [file 41598_2021_332_MOESM2_ESM.gif]

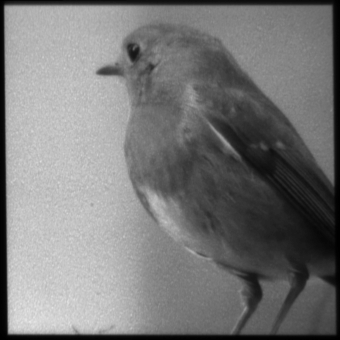

Supplement: Supplementary file 3 — Supplementary Information 3. [file 41598_2021_332_MOESM3_ESM.gif]

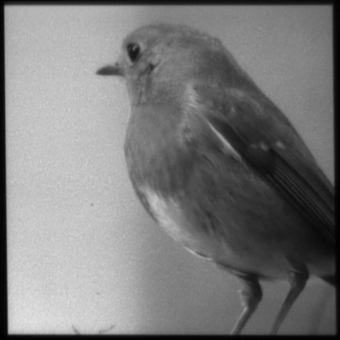

Supplement: Supplementary file 4 — Supplementary Information 4. [file 41598_2021_332_MOESM4_ESM.gif]

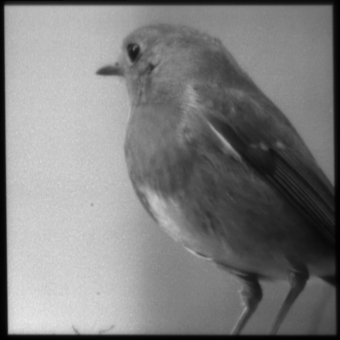

Supplement: Supplementary file 5 — Supplementary Information 5. [file 41598_2021_332_MOESM5_ESM.gif]

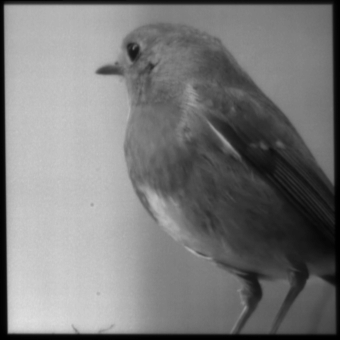

Supplement: Supplementary file 6 — Supplementary Information 6. [file 41598_2021_332_MOESM6_ESM.gif]
