# Supplementary material for: Artifact-free holographic light shaping through moving acousto-optic holograms
Source: Sci Rep. 2021 Oct 28;11:21261. doi: 10.1038/s41598-021-00332-4 (PMC8553788; doi:10.1038/s41598-021-00332-4)
Supplement: Supplementary file 7 — Supplementary Information 7. [file 41598_2021_332_MOESM7_ESM.docx]

Visualization 1: Experimental reconstruction of the video “Bird” (350x350 pixels) with a detector integration time of one hologram cycle τ=1T_T_ (τ=9.23 μs). The frame rate according to the integration time and the number of lines in each video frame was 310 Hz. A single hologram cycle is not sufficient to cancel out coherent artifacts, because one hologram cycles is required to fill the AOD aperture with the hologram, and another is needed to average out coherent artifacts. Therefore, degradations are observed which are for one part coherent artifacts, and for the other part crosstalk noise between the rapidly switching holograms that are displayed on the AOD.

Visualization 2: Experimental reconstruction of the video “Bird” (350x350 pixels) with a detector integration time of five hologram cycles τ=5T (τ=46.15 μs). The frame rate according to the integration time and the number of lines in each video frame was 62 Hz. The video frames show a significantly reduced crosstalk noise compared to the reconstruction obtained for an integration time of a single hologram cycle.

Visualization 3: Experimental reconstruction of the video “Bird” (350x350 pixels) with a detector integration time of eight hologram cycles τ=8T (τ=73.85 μs). The frame rate according to the integration time and the number of lines in each video frame was 39 Hz. The observed crosstalk noise is greatly reduced compared to the faster reconstructions. A large scale reconstruction of this video was perceived by the naked eye without flicker.

Visualization 4: Experimental reconstruction of the video “Bird” (350x350 pixels) with a detector integration time of ten hologram cycles τ=10T (τ=92.31 μs). The frame rate according to the integration time and the number of lines in each video frame was 31 Hz. The image quality increases with increasing detector integration times because the contribution of crosstalk noise reduces steadily.

Visualization 5: Experimental reconstruction of the video “Bird” (350x350 pixels) with a detector integration time of twenty hologram cycles τ=20T (τ=184.62 μs). The frame rate according to the integration time and the number of lines in each video frame was 15 Hz. The remaining crosstalk noise is hardly noticeable for these large integration times. Instead, the fluctuations of the laser beam power on a millisecond scale become visible, which result in fluctuating image line intensities.
